# Median Effective Volume of 0.2% Ropivacaine for Ultrasound‐Guided Axillary Brachial Plexus Block in Children Aged 6–10 Years: A Prospective Dose‐Finding Study Using Up‐and‐Down Sequential Allocation

**DOI:** 10.1002/pdi3.70049

**Published:** 2026-06-11

**Authors:** Tauseef Ahmad, Fei Yang, Yao Xu, Ying Yang, Huanjie Yun, Fei Xu, Yulin Xue, Yu Zhou, Zongke Shi, Yuan Shi, Shangyingying Li, Shengfen Tu

**Affiliations:** ^1^ Department of Anesthesiology, Children's Hospital of Chongqing Medical University, Ministry of Education Key Laboratory of Child Development and Disorders, Chongqing Key Laboratory of Child Neurodevelopment and Cognitive Disorders National Clinical Research Center for Child Health and Disorders Chongqing China

**Keywords:** axillary brachial plexus block, median effective volume, pediatric anesthesia, ropivacaine, ultrasound‐guided

## Abstract

The optimal dose for ultrasound‐guided axillary brachial plexus block (ABPB) in children remains poorly defined. This study aimed to find the median effective volume (EV_50_) of ropivacaine (0.2%) for ABPB in children aged 6–10 years. A prospective, double‐blind, dose‐finding trial using the Dixon up‐and‐down method was conducted. Pediatric patients (aged 6–10 years, ASA I–II) undergoing unilateral elbow or below‐elbow surgery were enrolled. Ultrasound‐guided ABPB was performed using a dual‐injection technique with supplemental musculocutaneous nerve blockade. The initial volume of ropivacaine (0.2%) was 0.5 mL/kg, adjusted by 0.05 mL/kg based on the previous patient's response. The study continued until seven inflection points were observed from failure to success. EV_50_ and EV_95_ (95% effective volume) were calculated using isotonic regression and bootstrapping, with 95% confidence intervals (CIs). Patient demographics, postoperative pain scores, and adverse events were monitored. Twenty‐nine patients were enrolled. The EV_50_ was 0.350 mL/kg (95% CI: 0.197–0.362 mL/kg), and the EV_95_ was 0.395 mL/kg (95% CI: 0.385–0.396 mL/kg). No adverse events were observed during the perioperative period. This study provides evidence‐based dosing parameters for 0.2% ropivacaine in pediatric ABPB, aiding clinicians in optimizing efficacy and safety. The findings support age‐specific precision in pediatric regional anesthesia.

## Graphical abstract

*Purpose*: This study aimed to determine the median effective volume (EV_50_) of 0.2% ropivacaine for ultrasound‐guided axillary brachial plexus block (ABPB) in children aged 6–10 years, using the Dixon up‐and‐down method. The EV_50_ was 0.350 mL/kg (95% CI: 0.197–0.362 mL/kg), calculated by isotonic regression and bootstrapping.

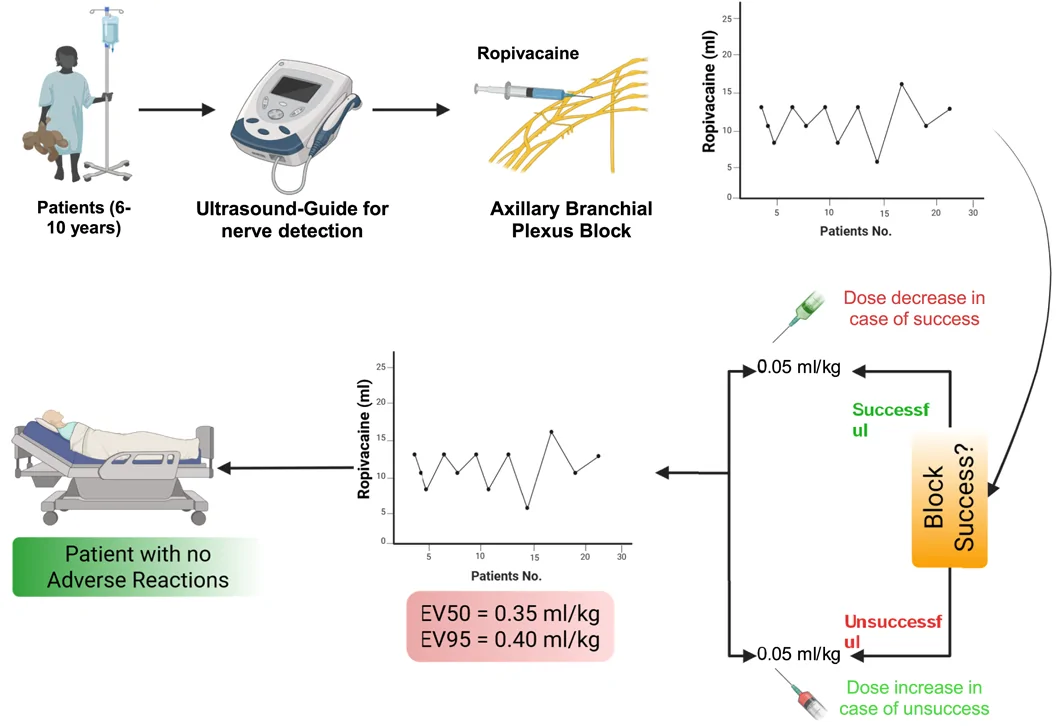

## 1 Introduction

Axillary brachial plexus block (ABPB) is one of the most commonly employed regional anesthesia techniques for pediatric upper extremity surgeries, providing notable benefits over general anesthesia alone, including enhanced postoperative analgesia and decreased opioid consumption [1]. This approach has seen substantial growth in pediatric anesthesia, especially with the broad integration of ultrasound guidance, which has markedly improved the accuracy and safety of peripheral nerve blocks in children [2]. Selecting suitable local anesthetic agents and dosing regimens continues to be a key factor in pediatric ultrasound‐guided ABPB.

Ropivacaine, a long‐acting amide local anesthetic, has become the agent of choice for pediatric brachial plexus blocks owing to its advantageous safety characteristics, lower cardiotoxicity relative to bupivacaine, and superior analgesic effects [3]. Joint guidelines from the European Society of Regional Anesthesia and Pain Therapy (ESRA) and the American Society of Regional Anesthesia and Pain Medicine (ASRA) advocate ropivacaine doses of 0.5–1.5 mg/kg for ultrasound‐guided upper extremity peripheral nerve blocks in children, highlighting the role of weight‐based dosing in maximizing efficacy and limiting toxicity [4].

The notion of minimum effective volume (MEV) has become increasingly important for refining regional anesthesia results. Emerging dose‐finding research has started to define evidence‐based dosing standards for pediatric groups. For instance, Chen et al. found that the EV_50_ and EV_95_ for 0.2% ropivacaine in ultrasound‐guided axillary brachial plexus block were 0.185 mL/kg and 0.280 mL/kg [1], respectively, in preschool‐aged children (3–6 years). Likewise, Liu et al. (2023) documented EV_50_ and EV_95_ values of 0.150 mL/kg and 0.195 mL/kg for supraclavicular brachial plexus blocks in children aged 1–6 years with the same ropivacaine concentration [5].

Existing data indicate that conventional weight‐based dosing may not yield ideal results across diverse pediatric age ranges. Up‐and‐down sequential allocation techniques, such as the Dixon method, have demonstrated utility in pinpointing exact effective doses for targeted groups, yielding more precise dosing guidance than generalized weight‐based methods [6]. The practical value of establishing accurate EV_50_ and EV_95_ extends past basic dosing refinement. These metrics equip clinicians with data‐driven resources to reduce local anesthetic use while preserving high success rates, thus lowering systemic toxicity risks and bolstering patient safety overall [7]. Moreover, uniform dosing protocols grounded in solid clinical data can mitigate the notable variations in current pediatric regional anesthesia practices. Drawing on prior studies and filling recognized research voids, this prospective investigation seeks to ascertain the EV_50_ and EV_95_ for 0.2% ropivacaine in ultrasound‐guided axillary brachial plexus block exclusively in children aged 6–10 years. The goal is to furnish clinicians with tailored local anesthetic dosing advice that enhances block effectiveness while curbing safety hazards in this distinct pediatric cohort.

## 2 Materials and Methods

### 2.1 Study Period and Location

This study was carried out from March to May 2025 at Children's Hospital, Chongqing Medical University in Chongqing, China.

### 2.2 Study Design

This prospective, double‐blind, dose‐finding clinical trial aimed to determine the EV_50_ and EV_95_ of 0.2% ropivacaine for ultrasound‐guided ABPB in children aged 6–10 years. The Dixon up‐and‐down sequential allocation methodology was employed, starting with an initial volume (0.5 mL/kg) of ropivacaine (0.2%). The volume for each consecutive patient was changed by 0.05 mL/kg based on the prior patient's response (success or failure). For ethical reasons, the minimal volume was chosen at 0.1 mL per kg. The study continued until seven inflection points (transitions from failure to success or vice versa) were observed.

### 2.3 Ethical Considerations

The Institutional Review Board of Children's Hospital of Chongqing Medical University approved the study protocol (approval number 161‐5/2020, approval date September 23, 2024). The trial was registered with the Chinese Clinical Trial Registry (ChiCTR2200057830; registration date: March 18, 2022. Primary Investigator: Dr. Li Yang). All participants' parents or legal guardians provided written informed consent, and participants aged 8 years and older completed their own written informed consent forms. The study followed the Declaration of Helsinki and Good Clinical Practice criteria.

### 2.4 Participants

Children aged 6–10 years with ASA physical status I–II were eligible for elective unilateral upper limb orthopedic trauma procedures involving the elbow or below. Exclusion criteria included anticoagulant therapy, infection at the puncture site, local anesthetic hypersensitivity, existing nerve injury or upper extremity paresthesia, intellectual disability, obesity, bilateral surgery, or refusal of consent by the patient or guardian.

### 2.5 Weight Calculation

The dosage of 0.2% ropivacaine was determined based on the Chinese growth and development standards for children under 6 years of age, excluding those with obesity, and was calculated using the children's actual body weight. To ensure dosing accuracy and patient safety, children with a body mass index (BMI) above the 95th percentile for sex and age were considered obese and were therefore excluded from the study, following the national standard WS/T 586‐2018 (National Health Commission of the People's Republic of China, 2018).

### 2.6 Anesthesia Procedures

Prior to surgery, intravenous access was established in the ward. Continuous monitoring in the operating room included electrocardiography (ECG), heart rate (HR), pulse oximetry (SpO_2_), respiratory rate (RR), temperature (T), and mean arterial pressure (MAP). Patients fasted for at least 6 hours for meals and 2 hours for clear fluids. Anesthesia was administered intravenously using midazolam (0.1 mg/kg, total dosage ≤ 2 mg), sufentanil (0.2 μg/kg), propofol (3 mg/kg), and penehyclidine hydrochloride (0.01 mg/kg). A propofol infusion was used to maintain anesthesia (5–10 mg/kg/h), titrated to keep bispectral index (BIS) readings between 40 and 60 [8]. Patients maintained spontaneous ventilation, and supplemental oxygen was administered using a face mask at a flow rate of 5 L/min.

### 2.7 Nerve Blocking Procedure

Ultrasound‐guided axillary brachial plexus block was carried out with the patient in the supine position, the ipsilateral arm abducted to 90°, and the elbow flexed. After aseptic skin preparation and draping, a high‐frequency linear array ultrasound probe (5.0–16.0 MHz, Venue 50, GE Medical Systems, China) was covered with a clean sleeve and inserted transversely in the axilla to identify the axillary artery and surrounding brachial plexus structures.

Under ultrasound guidance, the axillary artery appeared as an anechoic, pulsatile, circular structure. The cords and terminal branches of the brachial plexus, that is, the median, ulnar, and radial nerves, were identified and clustered around the artery, appearing as hypoechoic oval or round structures within the connective tissue sheath [9]. The musculocutaneous nerve was visualized separately, typically located within or adjacent to the coracobrachialis muscle. Color Doppler was used to confirm arterial pulsatility. Ultrasound images of the axillary approach are shown in Figure 1: (A) preinjection view showing perivascular nerves (ulnar, radial, and median) around the axillary artery and the musculocutaneous nerve; (B) needle placement for musculocutaneous nerve block; (C) postinjection spread of local anesthesia around the musculocutaneous nerve.

**FIGURE 1:**
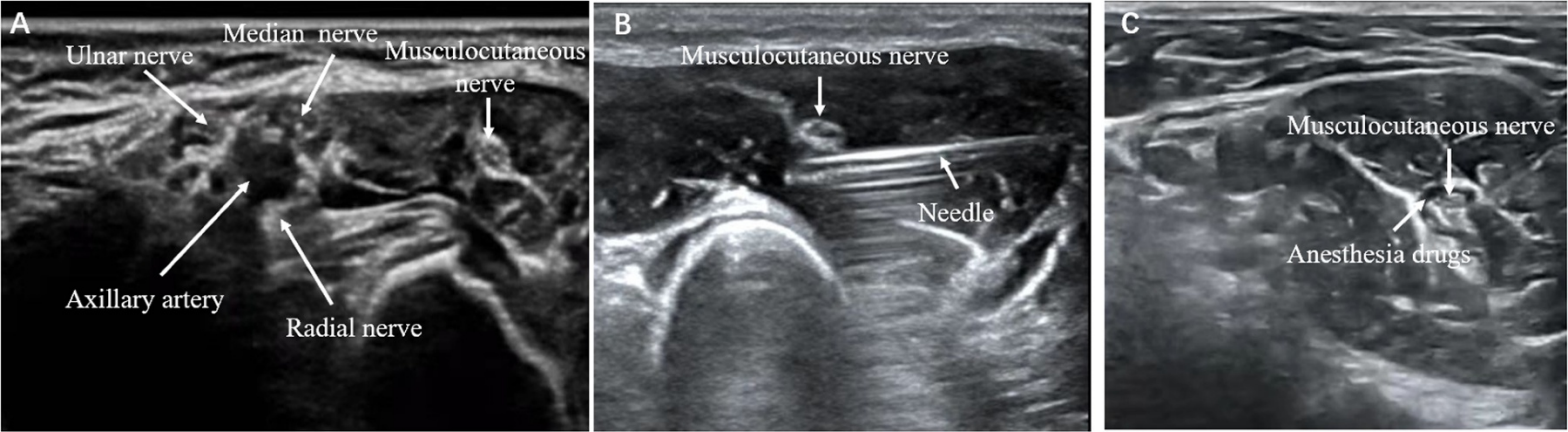
Ultrasound‐guided axillary brachial plexus block. (A) Preinjection view showing perivascular nerves (ulnar, radial, median) around the axillary artery and the musculocutaneous nerve. (B) Needle placement for musculocutaneous nerve block. (C) Postinjection spread of anesthetic drugs around the musculocutaneous nerve.

An echogenic needle with a short‐bevel tip (50 mm, 22 G Stimuplex D) was placed in‐plane and advanced under direct ultrasound guidance toward the brachial plexus. After negative aspiration to rule out intravascular implantation, the calculated volume of 0.2% ropivacaine was administered: approximately half targeting the positions at 6 and 12 o'clock around the axillary artery for median, ulnar, and radial nerve coverage, and 2 mL specifically for the musculocutaneous nerve (Figure 2) [10].

**FIGURE 2:**
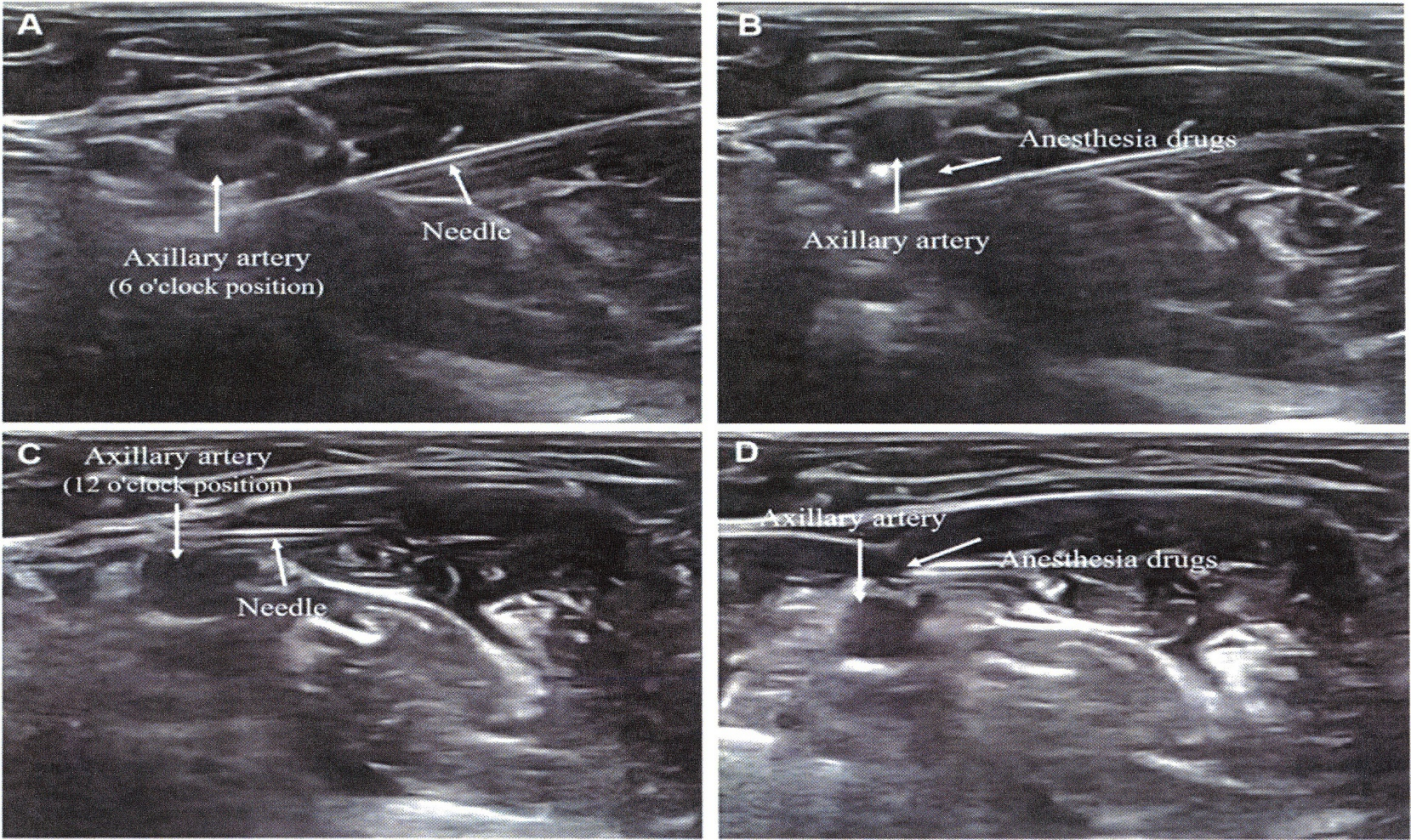
Ultrasound‐guided axillary brachial plexus block in children aged 6–10 years using a dual‐injection technique. (A) Preinjection needle placement at the 6 o'clock position relative to the axillary artery. (B) Postinjection spread of anesthetic drugs at the 6 o'clock position around the axillary artery. (C) Preinjection needle placement at the 12 o'clock position relative to the axillary artery. (D) Postinjection spread of anesthetic drugs at the 12 o'clock position around the axillary artery.

All procedures were carried out by an anesthesiologist with over 5 years of expertise. Postoperatively, children were monitored in the post‐anesthesia care unit (PACU), with discharge upon achieving a Steward score of ≥ 4 and being fully awake. Pain was measured using the modified Children's Hospital of Eastern Ontario Pain Scale (mCHEOPS), with scores > 6 requiring analgesia [11]. Adverse reactions, such as nerve injury, blood vessel damage, infection at the puncture site, agent poisoning, vomiting, dyspnea, and pneumothorax, were monitored for 3 days post‐surgery.

### 2.8 Surgical Timing

Surgery commenced 20 mins post‐block. Baseline HR and MAP were recorded 1 min before skin incision. If HR or MAP increased by > 20% from baseline or significant movement occurred, sufentanil (0.1–0.2 μg/kg) and propofol (1–2 mg/kg) were given. If unresolved or the patient experienced breathing difficulties, a laryngeal mask airway was placed or tracheal intubation was performed.

### 2.9 Data Collection

Baseline HR and MAP were recorded 1 min before incision [12]. Intraoperative block failure was defined as a > 20% increase in HR or MAP or significant patient movement, requiring supplemental propofol and sufentanil. Success was defined as a painless procedure with stable vital signs [11, 13]. Postoperatively, pain was assessed in the PACU using the mCHEOPS score, with scores > 6 requiring analgesia. The mCHEOPS evaluates five behavioral dimensions: cry, facial expression, verbal response, torso movement, and leg position. Each dimension is scored from 0 to 2, yielding a total score range of 0–10, where higher scores indicate greater pain. A total score > 6 suggests the need for analgesic intervention. This standardized tool has been validated for postoperative pain assessment in pediatric populations (Jeong et al., 2012) and was used in this study to ensure objective and reproducible evaluation of pain intensity [14]. Patients were discharged from the PACU with a Steward score of ≥ 4 [11, 14] and an mCHEOPS score of < 6. Adverse events (e.g., nerve injury, vascular damage, infection, local anesthetic toxicity, nausea, vomiting, and respiratory depression) were monitored for 3 days post‐surgery. In addition, all participants were contacted by telephone 1 week after surgery to assess any delayed adverse reactions. No delayed adverse events were identified, further confirming the safety and tolerability of the ropivacaine dosing regimen during short‐term follow‐up. Data comprised demographics (age, sex, height, weight, and ASA status), surgical side, operation time, resuscitation time, and adverse events (Table 1).

**TABLE 1:** Demographic, clinical, and operative characteristics stratified by success and failure of regional anesthesia in pediatric fracture surgery (*N* = 29).

Characteristic	Total (*n* = 29)	Success (*n* = 16)	Failure (*n* = 13)	*p*‐value
Demographics				
Male/female	20/9	11/5	9/4	1
Age (months)	93.07 ± 11.85	90.69 ± 13.59	96.00 ± 8.94	0.38
Weight (kg)	25.74 ± 4.65	25.59 ± 4.97	25.92 ± 4.23	0.85
BMI (kg/m^2^)	15.48 ± 1.72	15.60 ± 1.86	15.34 ± 1.60	0.69
Operative details				
Operation side (right/left)	15/14	9/7	6/7	N/A
Operation time (min)	57.21 ± 22.51	57.88 ± 22.05	56.38 ± 23.37	0.86
Anesthesia time (min)	96.07 ± 21.35	95.38 ± 21.20	96.92 ± 22.37	0.85
PACU time (min)	47.69 ± 14.53	44.69 ± 15.33	51.38 ± 13.13	0.22
Ropivacaine volume (mL/kg)	0.31 ± 0.08	0.34 ± 0.08	0.26 ± 0.06	**0.01**
Total dose of ropivacaine (mg)	7.94 ± 2.81	8.84 ± 3.15	6.85 ± 1.92	**0.04**
Complications				
Yes	0	0	0	N/A
No	29 (100%)	16 (100%)	13 (100%)	N/A
Surgical site				
Ulna	3 (10.3%)	3 (18.8%)	0 (0%)	0.26
Radius	4 (13.8%)	3 (18.8%)	1 (7.7%)	
Radius and ulna	4 (13.8%)	2 (12.5%)	2 (15.4%)	
Humerus	18 (62.1%)	8 (50.0%)	10 (76.9%)	
Preoperative diagnosis				
Humerus fracture	18 (62.1%)	8 (50.0%)	10 (76.9%)	0.27
Ulnar fracture	3 (10.3%)	3 (18.8%)	0 (0%)	
Radial fracture	4 (13.8%)	3 (18.8%)	1 (7.7%)	
Fracture of ulna and radius	4 (13.8%)	2 (12.5%)	2 (15.4%)	

*Note:* Data are presented as mean ± standard deviation, *n* (%), or *n*/*n* as appropriate. *p*‐values calculated based on independent *t*‐tests for continuous data and chi‐squared tests for categorical variables. Significant differences (*p* < 0.05) are highlighted in bold.

Abbreviations: BMI, body mass index; N/A, not applicable (no cases in subgroup); PACU, post‐anesthesia care unit.

### 2.10 Blinding

The ABPB was performed by an experienced anesthesiologist not involved in data collection or follow‐up. An independent observer, blinded to the ropivacaine volume, conducted intraoperative and postoperative assessments. Medication preparation was handled by a research assistant, and an operational assistant, also blinded, injected the anesthetic per instructions (e.g., “inject half”). Patients and guardians were unaware of the dose.

### 2.11 Study Termination Criteria

The study terminated after observing seven inflection points or if the minimum volume (0.1 mL/kg) was successful in five consecutive patients, ensuring a robust estimation of EV_50_.

### 2.12 Statistical Analysis

EV_50_ and EV_95_ were estimated using isotonic regression, with 95% confidence intervals (CI) derived via probit regression analysis (2000 bootstrap replicates). Pearson's correlation tested the relationship between ropivacaine volume and block duration. To identify predictors of block success, receiver operating characteristic (ROC) curve analysis was performed for each continuous variable: age, weight, BMI, ropivacaine volume (mL/kg), total ropivacaine dose (mg), duration of operation, duration of anesthesia, and duration of stay at PACU. For each variable, the area under the ROC curve (AUC) was calculated with 95% CI. The AUC quantifies a variable's ability to discriminate between success and failure, with interpretation as follows: AUC = 0.5 indicates no discriminative power (equivalent to random chance), 0.5–0.7 indicates poor discriminative power, 0.7 ≤ AUC < 0.8 indicates moderate discriminative power, and AUC ≥ 0.8 indicates strong discriminative power. The predicted determinants for block success were evaluated using ROC analysis [15, 16] (Table 2 and Figure 3). Data were analyzed with SPSS 27.0 and R 4.3.0. Categorical data are presented as frequencies and percentages, whereas continuous variables are represented as mean ± standard deviation.

**TABLE 2:** Receiver operating characteristic (ROC) analysis of predictive variables for successful regional anesthesia in pediatric fracture surgery.

Variable	AUC	95% CI	*p*‐value	Interpretation
Age	0.39	0.18–0.60	0.33	No predictive value
BMI	0.56	0.35–0.77	0.55	No predictive value
Weight	0.45	0.22–0.67	0.64	No predictive value
Duration of operation (min)	0.53	0.31–0.75	0.77	No predictive value
Duration of anesthesia	0.49	0.26–0.70	0.89	No predictive value
Duration of stay at PACU	0.36	0.15–0.56	0.20	No predictive value
Total dose of ropivacaine (mg)	0.70	0.52–0.89	0.06	No predictive value
Ropivacaine volume (mL/kg)	0.75	0.58–0.93	0.02*	Moderate‐to‐good discriminative power

*Note:* AUC interpretation: 0.5 means no discriminative power (random chance), 0.5–0.7 means poor discriminative power, 0.7–0.8 means moderate discriminative power, ≥ 0.8 means strong discriminative power. Statistical significance threshold: *p < 0.05. Only ropivacaine volume (mL/kg) showed significant predictive value (AUC 0.755, *p = 0.02). Analyses derived from a cohort of 29 pediatric patients.

Abbreviations: AUC, area under the curve; BMI, body mass index; CI, confidence interval; PACU, post‐anesthesia care unit.

**FIGURE 3:**
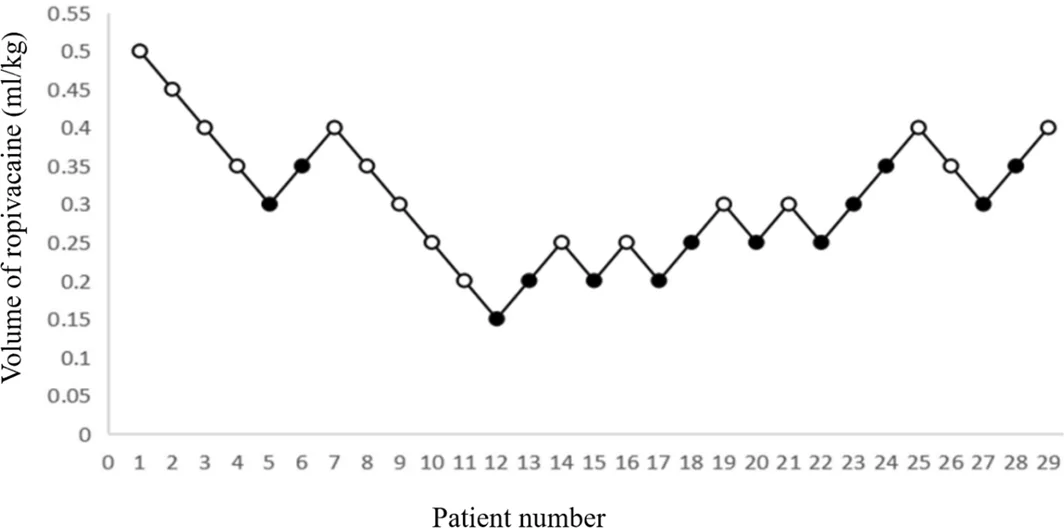
Diagram of the patient's block sequence (a solid circle represents the volume of failure, whereas an open circle represents the volume of success.) (*N* = 29).

## 3 Results

### 3.1 Ropivacaine Dosage Adjustment Protocol

The ropivacaine dosage adjustment procedure employed the Dixon up‐and‐down sequential allocation approach. For the patients aged 6–10 years, the initial dose was 0.5 mL/kg of 0.2% ropivacaine (Figure 4). After block administration, the axillary brachial plexus block was evaluated: If successful (Y path), the dose was decreased by 0.05 mL/kg for the subsequent patient; if unsuccessful (N path), it was increased by 0.05 mL/kg. This iterative process continued until seven convergence inflection points (transitions between success and failure) were observed, at which point the study terminated, and the EV_50_ was calculated as 0.350 mL/kg (95% CI: 0.197–0.362 mL/kg) and the EV_95_ as 0.395 mL/kg (95% CI: 0.385–0.396 mL/kg) via isotonic regression and bootstrapping.

**FIGURE 4:**
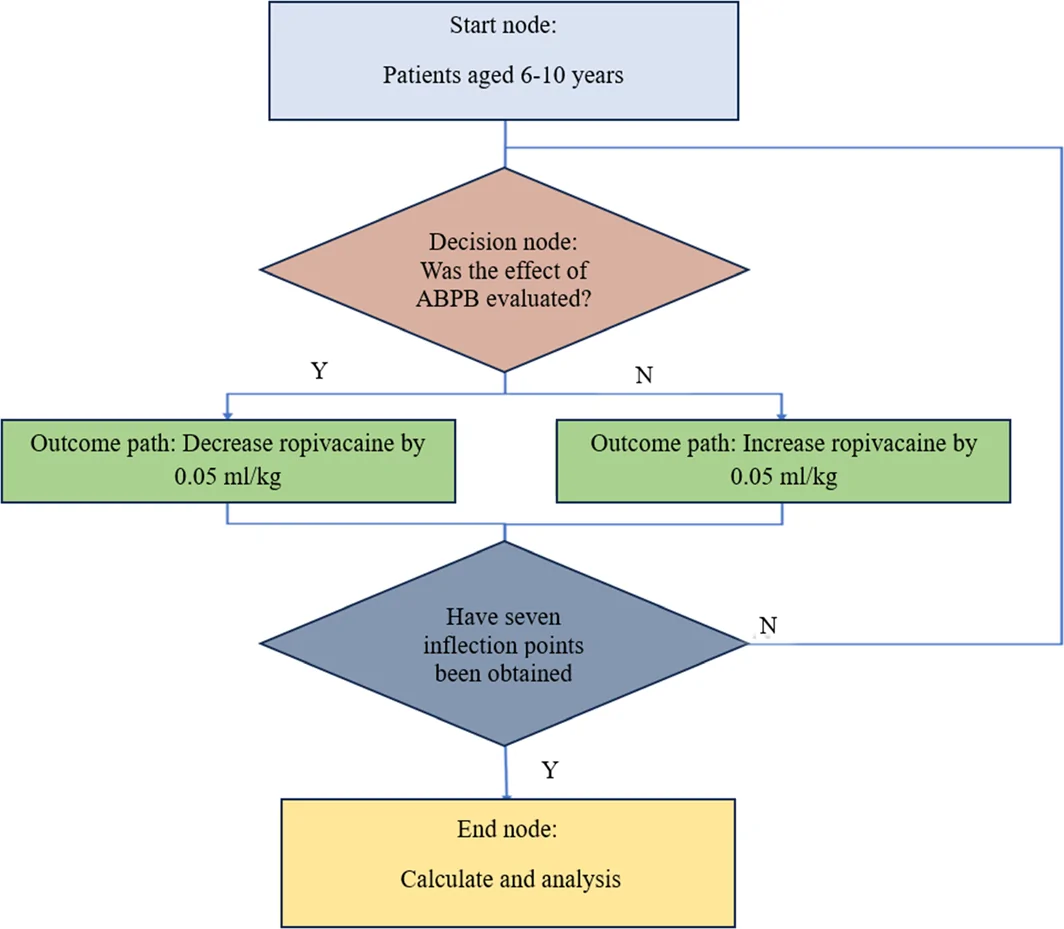
Algorithm for optimizing ropivacaine dosing in axillary brachial plexus blocks in children aged 6–10 years.

### 3.2 Demographics

Baseline demographics were well balanced between the successful (*n* = 16, 55.2%) and failed (*n* = 13, 44.8%) block groups (Table 1). The cohort included 20 males and 9 females (male‐to‐female ratio: 2.2:1), with a mean age of 93.07 ± 11.85 months and a mean weight of 25.74 ± 4.65 kg. No significant differences were found between groups in age (90.69 ± 13.59 vs. 96.00 ± 8.94 months, *p* = 0.38), weight (25.59 ± 4.97 vs. 25.92 ± 4.23 kg, *p* = 0.85), BMI (15.60 ± 1.86 vs. 15.34 ± 1.60 kg/m^2^, *p* = 0.69), or sex (*p* = 1.00), confirming group homogeneity.

### 3.3 Block Failure Details

Among the 13 patients labeled as “failed,” 8 exhibited significant vital changes (> 20% variation in vital parameters), whereas the remaining 5 demonstrated involuntary body movements. To facilitate painless surgery, sufentanil was administered for analgesia in these cases.

### 3.4 Sequential Block Success Pattern

The sequential allocation diagram (Figure 3) demonstrated a clear dose‐dependent efficacy trend, characterized by progressive convergence toward the median effective dose. Successful blocks predominantly clustered at higher volume levels, thereby substantiating the reliability and appropriateness of the up‐and‐down sequential allocation methodology in this dose‐finding context.

### 3.5 Dose–Response Analysis and Primary Endpoints

Successful blocks were associated with higher weight‐adjusted ropivacaine volumes (0.34 ± 0.08 vs. 0.26 ± 0.06 mL/kg, *p* = 0.01) and total doses (8.84 ± 3.15 vs. 6.85 ± 1.92 mg, *p* = 0.04). Probit regression estimated EV_50_ at 0.350 mL/kg (95% CI: 0.197–0.362 mL/kg) and EV_95_ at 0.395 mL/kg (95% CI: 0.385–0.396 mL/kg) (Table 3). The dose–response curve showed a sigmoidal pattern with a steep gradient between EV_20_ and EV_80_ (Figure 5).

**TABLE 3:** Dose–response estimates of ropivacaine for successful ultrasound‐guided regional anesthesia in children.

Parameter	Estimate (mL/kg)	95% CI	Interpretation
EV_50_	0.350	0.197–0.362	Median effective volume for 50% block success
EV_95_	0.395	0.385–0.396	Volume for 95% block success

*Note:* EV_50_, median effective volume (%) required for successful nerve block in 50% of patients; EV_95_, volume (%) required for 95% block success. Confidence intervals (95% CI) were derived from probit regression analysis. Data are based on observed outcomes in a cohort of 29 pediatric fracture surgery patients.

**FIGURE 5:**
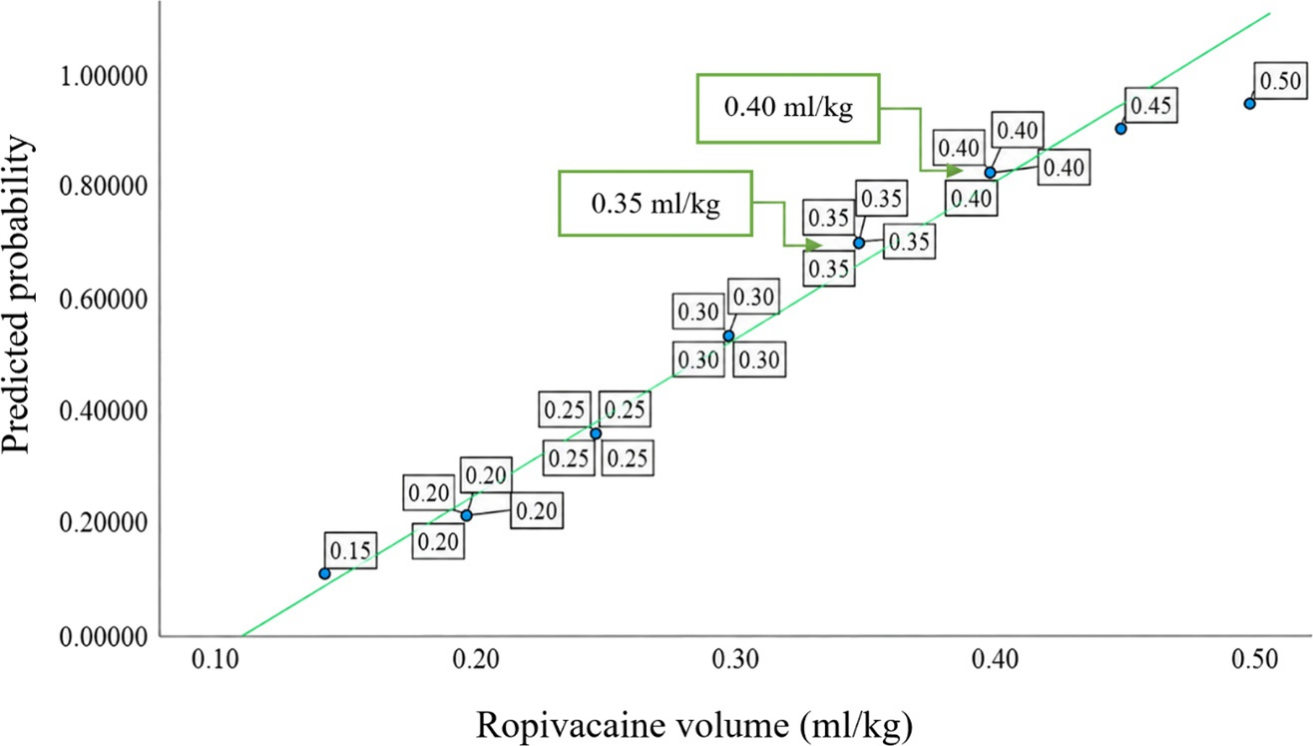
Dose–response curve for predicted probability of successful nerve block with ropivacaine. Data points represent observed outcomes at different dose levels, and the green line denotes the fitted dose–response curve based on a logistic regression model.

### 3.6 Predictive Performance Analysis

ROC analysis identified weight‐adjusted ropivacaine volume as the only significant predictor of success (AUC = 0.75, 95% CI: 0.58–0.93, *p* = 0.02; Table 2, Figure 6). Although the AUC for ropivacaine volume reached statistical significance, its value of 0.75 indicates moderate discriminative ability rather than strong predictive accuracy. This implies that, although ropivacaine volume represents a meaningful determinant of block success, other clinical or anatomical variables likely contribute to the observed outcomes. Hence, the ROC result should be interpreted as demonstrating a moderate, not exclusive predictive factor for successful regional anesthesia in this pediatric cohort. Demographic (age: AUC = 0.39, *p* = 0.33. BMI: AUC = 0.56, *p* = 0.55. Weight: AUC = 0.45, *p* = 0.64) and procedural (duration of operation: AUC = 0.53, *p* = 0.77. Duration of anesthesia: AUC = 0.49, *p* = 0.89. PACU time: AUC = 0.36, *p* = 0.20) variables showed no predictive value. Total ropivacaine dose trended toward significance (AUC = 0.70, *p* = 0.06).

**FIGURE 6:**
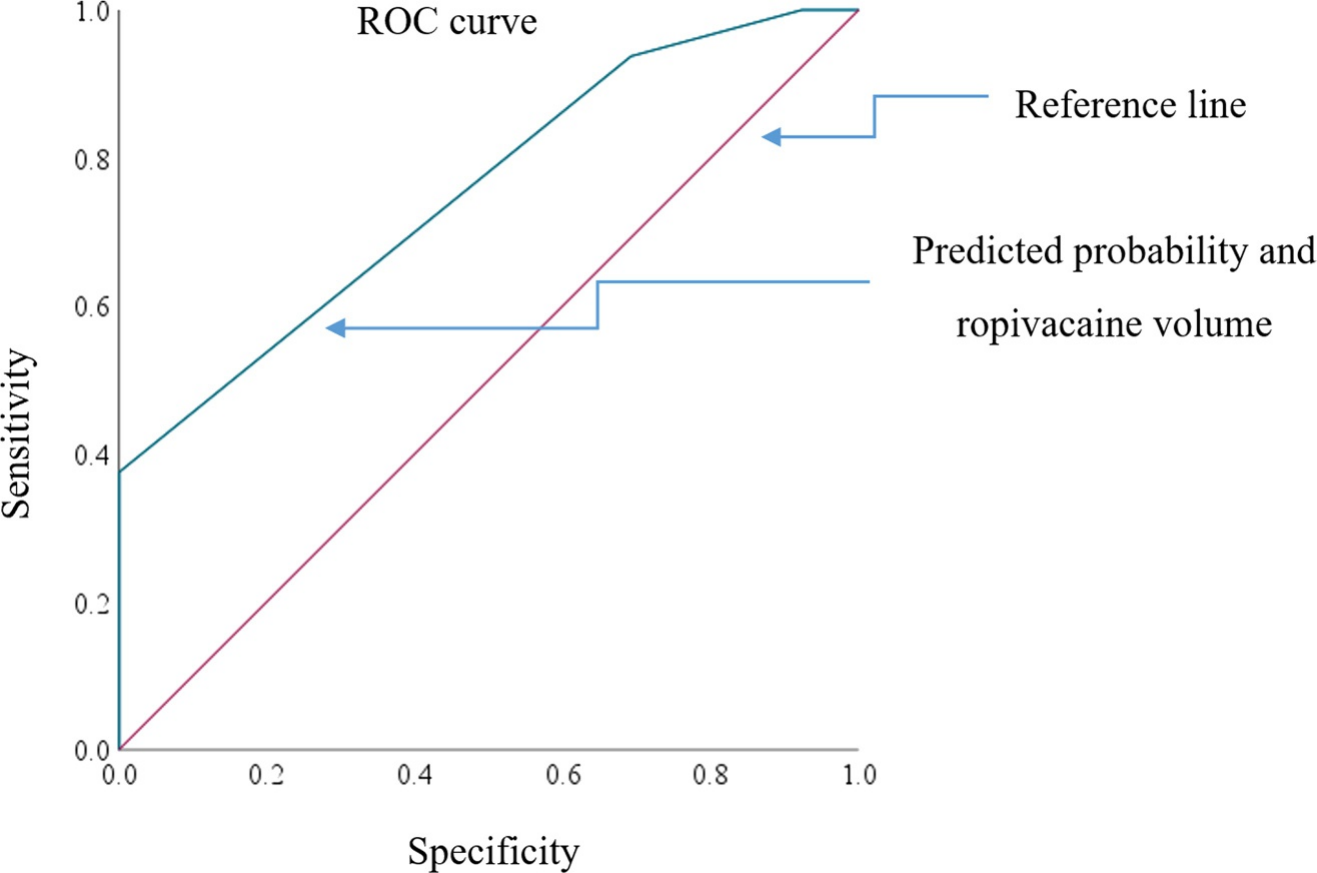
Ropivacaine receiver operating characteristic (ROC) curve for predicting successful pediatric regional anesthesia.

## 4 Discussion

Our results build upon prior investigations, including an EV_50_ of 0.185 mL/kg for ABPB in preschool‐aged children (3–6 years) [1]. This age‐related increase in ropivacaine requirement is consistent with our previous investigation on interscalene brachial plexus blocks in children aged 1–10 years [17], which reported a stepwise rise in the 90% minimum effective concentration (MEC_90_) of ropivacaine from 0.104% in toddlers to 0.133% in school‐age children. These findings support the general dose–age correlation pattern across different brachial plexus block approaches, reinforcing the concept that developmental anatomical and physiological maturation necessitates progressively higher effective concentrations in older pediatric populations. In addition, differences in block technique likely contribute. In our study, we adopted a dual‐injection approach at the 6 o'clock and 12 o'clock positions around the axillary artery, combined with a separate musculocutaneous nerve block, which ensures more uniform anesthetic distribution and reduces block failure [18]. Another investigation reported an EV_50_ of 0.150 mL/kg for supraclavicular blocks in children aged 1–6 years [5], further underscoring the influence of both age‐related anatomical factors and injection strategy on the required effective volume [19, 20].

The modestly higher effective volumes observed in our cohort may be explained by developmental differences in older children, including greater body size, thicker nerve sheaths, and denser connective tissue, emphasizing the need for age‐stratified rather than uniform pediatric dosing strategies [1, 21]. The 95% CI for the EV_50_ (0.197–0.362 mL/kg) observed in our study is relatively wide, which reflects the statistical precision of the estimate derived from the Dixon up‐and‐down sequential allocation design. This methodology inherently produces wider CIs than large cohort studies because it estimates effective doses using limited crossover events rather than large sample averages. Similar CI widths have been reported in other pediatric dose‐finding studies utilizing this approach [1, 5]. Importantly, despite this statistical dispersion, the close proximity between EV_50_ (0.350 mL/kg) and EV_95_ (0.395 mL/kg), and the absence of any adverse events or failed blocks at volumes ≥ 0.35 mL/kg, indicate that the dose–response curve is steep and the effective dosing range is narrow and clinically reliable. Thus, although the confidence interval width reflects the expected statistical properties of the study design, the clinical interpretation remains robust. In contrast, adult ABPB studies have reported minimum effective volumes of 0.5–1.0 mL/kg across concentrations [22], which are higher than our findings in children. This disparity likely reflects smaller anatomical compartments and superior ultrasound resolution in children owing to shallower tissue depths. For context, adult supraclavicular EV_50_ values of about 23 mL equate to 0.3–0.4 mL/kg when adjusted for weight [22], aligning with our results while accommodating pediatric nerve anatomy and distribution variances.

Despite advances in pediatric regional anesthesia, dosing uncertainties persist, often stemming from adult extrapolations or sparse child‐specific data leading to heterogeneous practices [21]. Pediatric Regional Anesthesia Network (PRAN) analyses indicate that up to 25% of children receive potentially supratherapeutic doses, underscoring the importance of minimizing local anesthetic volume to reduce systemic toxicity [21, 23]. Walker et al. reported that local anesthetic systemic toxicity occurred in only 0.76 per 10,000 pediatric regional anesthesia procedures, with the majority of cases involving higher‐risk neuraxial techniques rather than peripheral nerve blocks [23]. Ropivacaine, with its lower cardiotoxicity relative to bupivacaine due to reduced lipophilicity and sodium channel binding, is well suited for pediatric uses [24].

Our weight‐based approach aligns with ESRA/ASRA recommendations limiting ropivacaine to 0.5–1.5 mg/kg for pediatric peripheral blocks [4], minimizing exposure while achieving high success rates. Ultrasound guidance enables precise, low‐volume injections, that systematic reviews have shown to reduce anesthetic requirements, enhance block success, and lower complication rates compared with landmark‐based techniques [4, 7, 25]. Real‐time imaging facilitated targeted dual injections at the 6 and 12 o'clock positions around the axillary artery, contributing to our narrow CIs and circumferential spread confirmation. As emphasized by Di Filippo et al., this technique supports minimum effective volume strategies, which are crucial in children where immature metabolism and protein binding narrow the therapeutic window [18]. Consistent with these findings, our study employed ultrasound‐guided axillary block using a minimum effective volume strategy, and no adverse events were observed in any of the children.

ROC analysis identified weight‐adjusted ropivacaine volume as the only significant predictor of block success (AUC = 0.75, 95% CI: 0.58–0.93, *p* = 0.02). An AUC value of 0.75 indicates moderate discriminative ability rather than strong predictive accuracy; it confirms that ropivacaine volume represents a meaningful determinant of block success in this population. The absence of predictive value for demographic variables such as age, weight, and BMI, suggests that, within the narrow range of this study cohort (6–10 years, normal BMI), these factors do not independently influence block outcomes when weight‐adjusted dosing is employed.

### 4.1 Study Limitations

This study has several limitations. First, the focus on children aged 6–10 years limits extrapolation to other pediatric age groups, particularly infants and adolescents, who may have different anatomical and physiological characteristics. Second, the sample size (*n* = 29), although appropriate for the Dixon up‐and‐down methodology, may constrain precision for detecting infrequent adverse events or subgroup differences. Third, concurrent general anesthesia with propofol and sufentanil might have masked subtle signs of inadequate blockade, potentially influencing the classification of block success or failure; therefore, our findings represent dosing specifically under conditions of intravenous anesthesia with preserved spontaneous breathing. Fourth, the estimated effective volumes apply specifically to the dual‐injection perivascular technique with separate musculocutaneous nerve blockade and may not be generalizable to alternative approaches such as single‐injection or perineural techniques. Fifth, all enrolled patients had normal body weight (BMI below 95th percentile for age and sex), whereas obesity is increasingly common in clinical practice and may alter local anesthetic pharmacokinetics and distribution due to increased adipose tissue mass and changes in regional blood flow. Consequently, the applicability of our results to obese children remains limited and warrants separate investigation. Finally, the study was conducted at a single center, which may limit generalizability to other institutions with different patient populations or practice patterns.

## 5 Conclusion

This study establishes evidence‐based parameters for 0.2% ropivacaine in ultrasound‐guided axillary brachial plexus blocks in children aged 6–10 years, determining the EV_50_ and EV_95_. These findings advance precision medicine in pediatric regional anesthesia by providing age‐specific dosing targets that address knowledge gaps and reduce practice variations. The identification of weight‐adjusted volume as a statistically significant but moderately predictive factor (AUC = 0.75) supports its clinical relevance in guiding safe dosing within this age group; however, further research integrating additional physiological and procedural parameters is warranted to enhance predictive precision and optimize individualized dosing protocols.

## Author Contributions

T.A. and S.L. helped design and perform the study, and contributed significantly to the analysis and manuscript preparation. S.T. and F.Y. conceived, designed and funded the study. Y.X., Y.Y., H.Y., and F.X. performed the quality assessment. Y.X. helped to perform statistical analyses and search strategies. Y.Z., Z.S., Y.S., S.L., F.Y., and T.A. drafted and helped to perform the study. All authors have read and approved the manuscript.

## Funding

This study was funded by the National Clinical Research Center for Child Health and Disorders General Program of Clinical Medical Research (NCRCCHD‐2022‐GP‐0X) and the Natural Science Foundation of Chongqing Municipality (CSTB2023NSCQ‐MSX0434).

## Ethics Statement

The Institutional Review Board of Children's Hospital of Chongqing Medical University granted ethics approval for this study (approval number: [161‐5]/2020, approval date: Sep/23/2024). All procedures involving human volunteers in this investigation were carried out in compliance with ethical standards, including the principles outlined in the Declaration of Helsinki.

## Consent

Prior to participating in this study, all individuals' parents or legal guardians provided written informed consent. Participants aged 8 years and older completed their own written informed consent forms. The consent process includes full explanations of study procedures, potential hazards and benefits, and the option to withdraw at any time without penalty. This consent process is explicitly mentioned in the manuscript, and supporting documentation can be provided upon request if needed.

## Conflicts of Interest

The authors declare no conflicts of interest.

## Data Availability

The data supporting the study's conclusions are available from the corresponding author upon reasonable request.
